# Early Inflammatory Markers Are Independent Predictors of Cardiac Allograft Vasculopathy in Heart-Transplant Recipients

**DOI:** 10.1371/journal.pone.0113260

**Published:** 2014-12-09

**Authors:** Carlos A. Labarrere, John R. Woods, James W. Hardin, Beate R. Jaeger, Marian Zembala, Mario C. Deng, Ghassan S. Kassab

**Affiliations:** 1 CBL Partners for Life, Indianapolis, Indiana, United States of America; 2 California Medical Innovations Institute, San Diego, California, United States of America; 3 Methodist Research Institute at Indiana University Health and Richard M. Fairbanks School of Public Health, Indiana University-Purdue University, Indianapolis, Indiana, United States of America; 4 University of South Carolina, Columbia, South Carolina, United States of America; 5 Dr. Stein und Kollegen, Mönchengladbach, Germany; 6 Silesian Center for Heart Diseases, Zabrze, Poland; 7 Ronald Reagan UCLA Medical Center, Los Angeles, California, United States of America; 8 California Medical Innovations Institute, San Diego, California, United States of America; Center for Translational Medicine, United States of America

## Abstract

**Background:**

Identification of risk is essential to prevent cardiac allograft vasculopathy (CAV) and graft failure due to CAV (GFDCAV) in heart transplant patients, which account for 30% of all deaths. Early CAV detection involves invasive, risky, and expensive monitoring approaches. We determined whether prediction of CAV and GFDCAV improves by adding inflammatory markers to a previously validated atherothrombotic (AT) model.

**Methods and Findings:**

AT and inflammatory markers interleukin-6 (IL-6) and C-reactive protein (CRP) were measured in heart biopsies and sera of 172 patients followed prospectively for 8.9±5.0 years. Models were estimated for 5- and 10-year risk using (1) the first post-transplant biopsy only, or (2) all biopsies obtained within 3 months. Multivariate models were adjusted for other covariates and cross-validated by bootstrapping. After adding IL-6 and CRP to the AT models, we evaluated the significance of odds ratios (ORs) associated with the additional inflammatory variables and the degree of improvement in the area under the receiver operating characteristic curve (AUROC). When inflammatory markers were tested alone in prediction models, CRP (not IL-6) was a significant predictor of CAV and GFDCAV at 5 (CAV: p<0.0001; GFDCAV: p = 0.005) and 10 years (CAV: p<0.0001; GFDCAV: p = 0.003). Adding CRP (not IL-6) to the best AT models improved discriminatory power to identify patients destined to develop CAV (using 1^st^ biopsy: p<0.001 and p = 0.001; using all 3-month biopsies: p<0.04 and p = 0.008 at 5- and 10-years, respectively) and GFDCAV (using 1^st^ biopsy: 0.92 vs. 0.95 and 0.86 vs. 0.89; using all 3-month biopsies: 0.94 vs. 0.96 and 0.88 vs. 0.89 at 5- and 10-years, respectively), as indicated by an increase in AUROC.

**Conclusions:**

Early inflammatory status, measured by a patient's CRP level (a non-invasive, safe and inexpensive test), independently predicts CAV and GFDCAV. Adding CRP to a previously established AT model improves its predictive power.

## Introduction

Cardiac allograft vasculopathy (CAV), an aggressive form of atherosclerosis, is the leading cause of graft failure in heart-transplant patients surviving beyond the first year [1] and is responsible for up to 30% of all deaths [2]. CAV is similar in many respects to native coronary artery disease (CAD). Unlike native CAD, however, which takes a lifetime to develop, CAV occurs very rapidly, within months to a few years after transplantation and develops uniformly throughout the entire vasculature. Because of its rapid occurrence, early detection is critical to the successful management of transplant patients. Thus, research has focused on identifying biomarkers that can reliably predict future CAV and graft failure.

We have shown previously that atherothrombotic (AT) markers detectable very early in biopsied heart tissue are reliably associated with CAV development and graft failure. These markers include the expression of intercellular adhesion molecule-1 (ICAM-1) [3]–[5], the presence of fibrin [6], [7], and the loss of microvascular antithrombin [8] and tissue plasminogen activator (tPA) [9].

Recently, we tested all of these AT markers in risk prediction models and demonstrated that Graft Failure Due to CAV (GFDCAV) very rarely develops in patients who show early absence of fibrin within 9 days post-transplantation (negative predictive accuracy using a single biopsy: 99% at 5 years and 96% at 10 years) [10], and persistence of normal tPA levels over the next 3 months (negative predictive accuracies: 99% at 5 years and 95% at 10 years) [10], [11]. This finding is clinically significant, implying that it is possible to identify a subgroup of patients within weeks of transplantation that may be able to safely forgo intensive monitoring with serial biopsies, a common practice in most transplant centers that is expensive and carries risks for patients.

Since CAV is also associated with systemic inflammation, as measured by elevated serum C-reactive protein (CRP) levels [3], [12], we sought to determine in the present study whether a patient's inflammatory status is independently predictive of CAV and GFDCAV and whether adding inflammatory status to our previously established AT models would significantly improve the model's predictive value.

## Methods

### Patients

The study population consisted of 241 consecutive adult patients with hearts transplanted from August 1989 to August 2004. Patients were included in the analysis if they survived at least three months after transplantation, had serial endomyocardial biopsies performed in the first three months, and had their coronary arteries examined angiographically and/or histopathologically for CAV at annual follow-ups. Of the original 241 candidates, 69 patients were excluded from analysis for the following reasons: 29 patients were missing three-month biopsy data, either because they died prior to three months (n = 14) or because transplantation occurred at another institution (n = 15); 38 survived three months but were excluded because of incomplete biopsy data; two survived but were excluded because of missing follow-up coronary evaluations. This left a sample of 172 patients who were followed prospectively until September 2010 (mean follow-up: 8.9±5.0 years). The Indiana University local Institutional Review Board approved the study protocol and all subjects signed a consent form.

Clinical management and outcome criteria have been previously described by Labarrere et al [10]. Endomyocardial biopsies were performed on all 172 donor hearts at the time of transplantation before reperfusion (baseline) and serially during the first three months, with the first post-transplant biopsy obtained within a median 9 days of transplantation. CAV was evaluated in annual angiograms (mean number per patient: 5.25±1.0). CAV was diagnosed if there were evidence of narrowing or luminal irregularities either in the left main or any primary or branch coronary vessels. CAV was determined by consensus of two experienced angiographers unaware of IL-6 and CRP levels or biopsy data. For recipients who died before their first annual angiogram, coronary arteries were examined histopathologically and GFDCAV was defined as (a) death associated with CAV-related cardiac allograft dysfunction, or (b) need for a second transplant due to severe CAV (left main stenosis>70%, two or more primary vessels with stenoses>70%, or branch stenoses>70% in all three systems) [8].

### Determination of IL-6 and CRP concentrations

Serial serum samples, obtained at the time of each endomyocardial biopsy, were stored at −75°C for later analysis. Samples were thawed and assayed in duplicate for IL-6 and CRP using enzyme-linked immunosorbent assays (Human IL-6, Quantikine HS [high sensitivity] 600B, R&D Systems, Minneapolis, MN, USA; human CRP, ADI, San Antonio, TX, USA). Minimum detectable concentrations of IL-6 and CRP were 0.039 pg/mL and 350 pg/ml, respectively. Laboratory personnel were unaware of biopsy data or patients' outcome.

### Immunohistochemistry studies

Endomyocardial biopsies were tested for fibrin, tPA, antithrombin, and ICAM-1 as described by Labarrere et al [10]. Immunohistochemical data were evaluated by two investigators unaware of clinical outcomes.

### Coding of biomarkers

**Figure 1:**
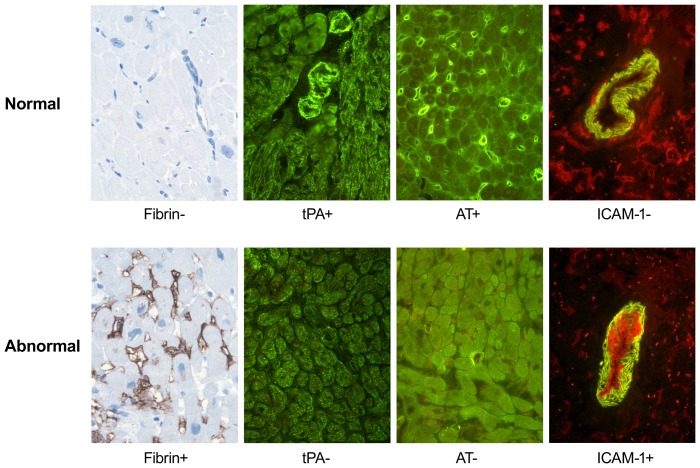
Immunohistochemical characteristics of a thrombotic/activated microvasculature. Normal hearts (top row) have absence of fibrin (Fib-), presence of microvascular antithrombin (AT+) and tissue plasminogen activator (tPA+) and absence of arterial endothelial ICAM-1 (ICAM-1-). Abnormal thrombotic and activated hearts (bottom row) are characterized by presence of fibrin (Fib+), loss of microvascular antithrombin (AT-) and tissue plasminogen activator (tPA-) and expression of arterial endothelial ICAM-1 (ICAM-1+). Original magnification ×640. This figure has been reproduced with permission from: Labarrere CA, Woods JR, Hardin JW, Campana GL, Ortiz MA, et al. (2012) Value of the First Post-Transplant Biopsy for Predicting Long-Term Cardiac Allograft Vasculopathy (CAV) and Graft Failure in Heart Transplant Patients. PLoS ONE 7(4): e36100. doi: 10.1371/journal.pone.0036100.

For predictive modeling, immunohistochemical data were scored as described by Labarrere et al [11] as illustrated in **Fig. 1**. For models that used only the first biopsy, immunohistochemical signs were scored either 0 (normal) or 1 (abnormal). For models that used all biopsies, the *proportion* of abnormal signs for each marker was calculated and re-scaled by a factor of 10 so that regression coefficients could be interpreted in terms of a 10% change in the proportion of abnormal biopsies. For IL-6 and CRP, median values obtained from all samples acquired in the first 3-months post-transplant for each patient were used in all predictive models.

### Statistical models

Separate logistic regression models were developed to predict the onset of CAV and GFDCAV at 5 and 10 years after transplantation. These models were estimated from information obtained from the first biopsy only, and then re-estimated using information from all biopsies available in the first 3 months after transplantation. We considered the four AT predictors (fibrin, antithrombin, tPA, ICAM-1) and the two inflammatory predictors (median IL-6 and CRP values measured over the first 3 months). We developed two sets of models: one considered only inflammatory status, ignoring information about AT risk factors: the second evaluated whether adding inflammatory status to a model that already contained AT risk predictors improves the model prediction.

For the first set of models, the inflammatory markers IL-6 and CRP were used as the sole predictors using each marker in turn in separate univariate regressions; then both markers were included together in a multivariate equation.

For the second set of models we added the inflammatory markers, IL-6 and CRP, to our previously reported prediction models [10], [11], which included only atherothrombotic markers but not inflammatory markers. We refer to the previously reported atherothrombotic-only models as the “Established Models.” To determine whether knowledge of inflammatory status contributes additional predictive value to the Established Models, we re-estimated the Established Models with either IL-6, CRP, or both included as additional inflammatory predictors. We refer to these re-estimated models, which included both atherothrombotic as well as inflammatory markers, as the “New Models.”

Coefficients associated with the atherothrombotic markers and adjustment variables from the Established Models were constrained in the re-estimated models so that we could determine whether adding inflammatory status significantly improved predictive accuracy beyond that already achieved by knowing the patient's atherothrombotic status. The incremental contribution to predictive accuracy was evaluated by noting whether the odds ratios (ORs) associated with the inflammatory markers were independently predictive of outcome, and by comparing the Established and New Models in terms of overall improvement in the AUROC, and gains in the percentage of correctly classified patients [13].

Effron's bootstrap method [14] was used to validate model coefficients. Each model was estimated 200 times using repeated samples drawn from the original data with replacement. We calculated receiver operating characteristic (ROC) curves and used the Youden Index [15] to identify optimum cut-off values. Model performance was further quantified by evaluating sensitivity, specificity, and predictive accuracy. The AUROC [16] was used to quantify the models' discriminative accuracy. Bootstrapping of 1,000 repeated samples was used to test the statistical significance of improvements in the AUROC attributable to adding inflammatory markers to the model. We also compared overall percentages of patients correctly classified.

## Results

Demographic and clinical characteristics of the patient population assessed at three months post-transplant are shown in **Table 1**.

**Table 1:** Summary of demographic and clinical variables (Patients: n = 172).

VARIABLE	VALUE					
**Donor:**						
Age, mean years (±SD)	28.8					(±11.2)
Sex (percent male)	78.5					
**Recipient:**						
Age, mean years (±SD)	48.7					(±10.2)
Sex (percent male)	66.9					
Race (percent white)	89.5					
Body mass index (kg/m^2^), mean (±SD)	26.5					(±5.0)
Diabetics (%)	40.1					
Insulin dependent diabetics (%)	31.4					
**Reason for transplantation:**						
Coronary artery disease (%)	45.9					
Cardiomyopathy (%)	47.1					
Other (%)	7.0					
**Ischemic time (minutes), mean (±SD)**	156.8					(±56.6)
**Smokers after transplantation (%)**	7.6					
**Hypertensives (%)**	89.0					
**Cholesterol (mmol/l):**						
Total cholesterol, mean (±SD)	5.4					(±1.0)
LDL-C, mean (±SD)	2.6					(±0.8)
HDL-C, mean (±SD)	1.2					(±0.4)
**Number of HLA mismatches:**	0	1	2	3	4	
A/B (%)	0	5.8	16.3	47.1	30.8	
DR (%)	7.00	39.0	54.0			
**Creatinine>123.8 µmol/l (%)**	58.1					
**Ejection fraction, mean (%) (±SD)**	54.3					(±7.4)
**2R-3R rejections (1^st^ 3-mos), mean (±SD)**	0.2					(±0.4)
**Biopsies (1^st^ 3-months), mean (±SD)**	5.2					(±1.0)
**CMV infections (% positive)**	12.8					
**Cell Panel Reactive Antibodies>0% (%)**	8.1					
**Treatment:**						
Prednisone (%)	100.0					
Cyclosporine (%)	94.2					
Azathioprine (%)	68.0					
Mycophenolate mofetil (%)	65.7					
Tacrolimus (%)	11.0					
Sirolimus (%)	7.0					
Statins (%)	77.9					
Calcium Channel Blockers (%)	77.9					
ACE Inhibitors/ARBs (%)	43.0					

All data based on entire sample of 172 patients.

**Abbreviations**: SD: Standard Deviation; HLA: human leukocyte antigen; LDL-C: low density lipoprotein cholesterol; HDL-C: high density lipoprotein cholesterol; CMV: cytomegalovirus; ACE: Angiotensin-Converting Enzyme; ARB: Angiotensin Receptor Blockers.

### Predictive value when inflammatory markers are the sole predictors

Results for regression models using only inflammatory markers IL-6 and CRP to predict CAV and GFDCAV are provided in **Tables 2** **and** **3**. In univariate models, CRP was a significant predictor of CAV and GFDCAV at both 5 and 10 years post-transplant. In no case was IL-6 a significant univariate predictor. In multivariate models where both CRP and IL-6 were included together as predictors, CRP (but not IL-6) was significantly predictive of CAV at both 5- and 10-years post-transplant (**Table 2**) and GFDCAV at 10-years post-transplant (**Table 3**). IL-6 (but not CRP) was a marginally significant predictor (*P* = 0.06) of GFDCAV at 5 years (**Table 3**). Discriminative performance as measured by the AUROC ranged from 0.77 to 0.86.

**Table 2:** Logistic regression models using information from inflammatory markers IL-6 and CRP to predict CAV at 5- and 10-years post-transplantation.

	5-Year Risk of CAV				10-Year Risk of CAV			
	OR	*p*	95% C.I.	ROC Area	OR	*p*	95% C.I.	ROC Area
Univariate Models:								
IL-6, only	1.04	0.59	0.91–1.18	0.70	0.99	0.91	0.88–1.12	0.33
CRP, only	1.41	<0.0001	1.24–1.61	0.81	1.55	<0.0001	1.30–1.84	0.83
Multivariate Model:								
IL-6	1.90	0.13	0.83–4.34	0.84	1.34	0.44	0.64–2.81	0.83
CRP	1.21	0.02	1.03–1.40		1.51	<0.0001	1.26–1.82	

Median IL-6 and CRP values measured over the first 3 months post-transplant were used in all predictive models.

**Abbreviations**: IL-6: Interleukin-6; CRP: C-reactive protein; CAV: Cardiac allograft vasculopathy; OR: Odds ratio; C.I.: Confidence interval; ROC: Receiver operating characteristic.

**Table 3:** Logistic regression models using information from inflammatory markers IL-6 and CRP to predict GFDCAV at 5- and 10-years post-transplantation.

	5-Year Risk of GFDCAV				10-Year Risk of GFDCAV			
	OR	*p*	95% C.I.	ROC Area	OR	*p*	95% C.I.	ROC Area
Univariate Models:								
IL-6, only	1.04	0.63	0.89–1.20	0.75	1.01	0.92	0.87–1.17	0.66
CRP, only	1.19	0.005	1.05–1.34	0.86	1.16	0.003	1.05–1.28	0.80
Multivariate Model:								
IL-6	2.69	0.06	0.94–7.72	0.84	1.60	0.26	0.71–3.63	0.77
CRP	1.11	0.10	0.98–1.24		1.11	0.05	1.00–1.24	

Median IL-6 and CRP values measured over the first 3 months post-transplant were used in all predictive models.

**Abbreviations**: IL-6: Interleukin-6; CRP: C-reactive protein; graft failure due to cardiac allograft vasculopathy: GFDCAV; OR: Odds ratio; C.I.: Confidence interval; ROC: Receiver operating characteristic.

### Improvement in prediction when inflammatory markers are added to the Established Models

Regressions with IL-6 and CRP as additional predictors in the Established Models are summarized for the predictions of CAV (**Tables 4** **and** **5**) and GFDCAV (**Tables 6** **and** **7**). For each condition summarized in **Tables 4** **,** **5** **,** **6** **and** **7** **,** we estimated three different models that added either IL-6, CRP, or both to the Established Model. Results are reported for the best models as judged by the improvement in the AUROC. In nearly all cases, CRP (but not IL-6) was statistically significant when included in the Established Models, indicating that a patient's inflammatory status is independently predictive of outcome, as measured by elevated serum CRP concentrations, after accounting for statistically significant measures of the patient's atherothrombotic status.

**Table 4:** Logistic regression models for the prediction of CAV using the first biopsy obtained within a median 9-days of transplantation.

*First Biopsy*	5-Year Risk of CAV				10-Year Risk of CAV		
	OR	*p*	95% C.I.		OR	*p*	95% C.I.
*Established Model (AT, only):*				*Established Model (AT, only):*			
Antithrombin	5.34	<0.0001	2.69–10.58	Antithrombin	8.73	<0.0001	3.81–20.04
3-mo rejections	0.33	0.008	0.14–0.75	MMF regimen	0.37	0.02	0.16–0.84
				Recipient sex (male)	2.01	0.08	0.93–4.37
				HLA-AB mismatch	0.41	0.05	0.17–0.99
				Statins	2.12	0.11	0.85–5.30
*Inflammatory markers added:*				*Inflammatory markers added:*			
CRP	1.32	<0.0001	1.16–1.51	CRP	1.41	0.0002	1.18–1.69

Established Models use information from atherothrombotic markers, only (AT, only). New Models add information from inflammatory markers (CRP and IL-6) to the Established Model.

**Abbreviations**: CAV: Cardiac Allograft Vasculopathy; CRP: C-reactive protein; IL-6: Interleukin-6; OR: Odds Ratio; C.I.: Confidence Interval; AT: Atherothrombotic; MMF: Mycophenolate mofetil; HLA-AB: human leukocyte antigen-A, -B.

**Table 5:** Logistic regression models for the prediction of CAV using all biopsies obtained within the first 3-months post-transplant.

*All Biopsies in 1^st^ 3-Months*	5-Year Risk of CAV				10-Year Risk of CAV		
	OR	*p*	95% C.I.		OR	*p*	95% C.I.
*Established Model (AT, only):*				*Established Model (AT, only):*			
tPA	1.41	<0.0001	1.26–1.58	Antithrombin	1.47	<0.0001	1.29–1.68
Recipient sex (male)	2.34	0.04	1.05–5.19	MMF regimen	0.49	0.09	0.21–1.12
HLA-AB mismatch	0.37	0.02	0.15–0.88	Recipient sex (male)	2.55	0.03	1.12–5.80
				HLA-AB mismatch	0.48	0.12	0.19–1.20
				3-mo rejections	0.40	0.10	0.13–1.21
*Inflammatory markers added:*				*Inflammatory markers added:*			
CRP	1.25	0.002	1.09–1.44	CRP	1.34	0.002	1.11–1.61

Established Models use information from atherothrombotic markers, only (AT, only). New Models add information from inflammatory markers (CRP and IL-6) to the Established Model.

**Abbreviations**: CAV: Cardiac Allograft Vasculopathy; CRP: C-reactive protein; IL-6: Interleukin-6; OR: Odds Ratio; C.I.: Confidence Interval; AT: Atherothrombotic; tPA: tissue plasminogen activator; MMF: Mycophenolate mofetil; HLA-AB: human leukocyte antigen-A, -B.

**Table 6:** Logistic regression models for the prediction of GFDCAV using the first biopsy obtained within a median 9-days of transplantation.

*First Biopsy*	5-Year Risk of GFDCAV				10-Year Risk of GFDCAV		
	OR	*p*	95% C.I.		OR	*p*	95% C.I.
*Established Model (AT, only):*				*Established Model (AT, only):*			
Fibrin	9.33	0.001	2.34–37.12	Fibrin	3.99	0.005	1.53–10.40
MMF regimen	0.08	0.0008	0.02–0.35	MMF regimen	0.12	<0.0001	0.04–0.32
Statins	0.10	0.0007	0.28–0.38	Statins	0.21	0.002	0.08–0.55
*Inflammatory markers added:*				*Inflammatory markers added:*			
CRP	1.12	0.01	1.02–1.23	CRP	1.09	0.03	1.01–1.17

Established Models use information from atherothrombotic markers, only (AT, only). New Models add information from inflammatory markers (CRP and IL-6) to the Established Model.

**Abbreviations**: Graft Failure Due to Cardiac Allograft Vasculopathy: GFDCAV; CRP: C-reactive protein; IL-6: Interleukin-6; OR: Odds Ratio; C.I.: Confidence Interval; AT: Atherothrombotic; MMF: Mycophenolate mofetil.

**Table 7:** Logistic regression models for the prediction of GFDCAV using all biopsies obtained within the first 3-months post-transplant.

*All Biopsies in 1^st^ 3-Months*	5-Year Risk of GFDCAV				10-Year Risk of GFDCAV		
	OR	*p*	95% C.I.		OR	*p*	95% C.I.
*Established Model (AT, only):*				*Established Model (AT, only):*			
tPA	1.73	0.002	1.22–2.45	tPA	1.31	0.001	1.12–1.54
MMF regimen	0.11	0.004	0.25–0.49	MMF regimen	0.15	0.0002	0.06–0.41
Statins	0.06	0.0002	0.01–0.27	Recipient sex (male)	2.77	0.08	0.88–8.77
				Statins	0.17	0.0006	0.06–0.47
*Inflammatory markers added:*				*Inflammatory markers added:*			
CRP	1.11	0.03	1.01–1.21	CRP	1.09	0.04	1.01–1.18

Established Models use information from atherothrombotic markers, only (AT, only). New Models add information from inflammatory markers (CRP and IL-6) to the Established Model.

**Abbreviations**: Graft Failure Due to Cardiac Allograft Vasculopathy: GFDCAV; CRP: C-reactive protein; IL-6: Interleukin-6; OR: Odds Ratio; C.I.: Confidence Interval; AT: Atherothrombotic; tPA: tissue plasminogen activator; MMF: Mycophenolate mofetil.

**Figure 2:**
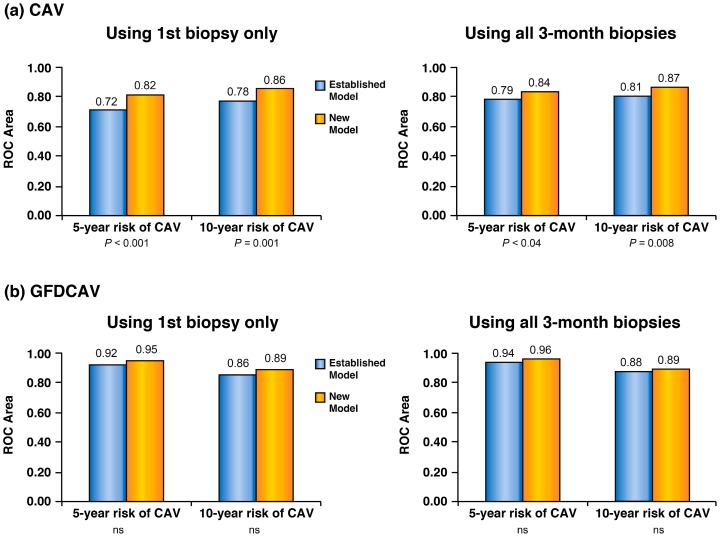
Prediction of (a) CAV and (b) Graft Failure Due to CAV (GFDCAV). Including inflammatory status (CRP) to the established AT prediction model improves discriminatory power as defined by the increase in the area under the ROC curve.

A common method of judging whether a new model represents an improvement over an established model is to compare the AUROC values of the two models [13]. Adding the best inflammatory predictors (usually CRP, alone) to the Established Model improved model discrimination in all cases (**Fig. 2**). Not all improvements, however, were statistically significant. The lack of statistical significance was primarily due to the Established Model's AUROC statistics already being quite high which leaves little margin for improvement.

**Tables 8** **and** **9** summarize other performance characteristics of the New Models. On the whole, sensitivity and specificity were improved, indicating that the New Models are better able to single out patients at risk of CAV and GFDCAV without falsely identifying those not at risk. For the clinician, an important question concerns the accuracy of a given patient's result, which is reflected in the model's positive and negative predictive value (PPV and NPV). For the prediction of CAV (**Table 8**), PPV and NPV improved in most instances when the patient's inflammatory status was added to the Established Model. For the prediction of GFDCAV (**Table 9**), NPV was already quite high in the Established Models and remained high when inflammatory status was added to the model. PPVs for the prediction of GFDCAV, however, were low and remained low, even after taking inflammatory status into account. Thus, heart transplant recipients exhibiting an early negative result, whether measured by the New Models or the Established Models, are very *unlikely* to develop GFDCAV over the next 10 years and can be reassured accordingly. However for those exhibiting early positive results, predictions are less reliable.

**Table 8:** CAV: Performance of the Established Model (which uses atherothrombotic markers, only), with the New Model (which adds inflammatory markers to the Established Model).

	Using 1^st^ biopsy, only		Using all 3-month biopsies	
***5-year risk***	*Established Model*	*New Model*	*Established Model*	*New Model*
Sensitivity	0.79	0.70	0.78	0.87
Specificity	0.58	0.84	0.70	0.63
PPV	0.65	0.81	0.72	0.70
NPV	0.74	0.73	0.76	0.83
Pct Correct^a^	0.69	0.77	0.74	0.75
Cutoff Value^b^	0.27	0.56	0.44	0.30
Prevalence^c^	0.50		0.50	
***10-year risk***				
Sensitivity	0.62	0.79	0.76	0.77
Specificity	0.82	0.83	0.75	0.79
PPV	0.86	0.89	0.84	0.87
NPV	0.56	0.70	0.64	0.67
Pct Correct^a^	0.70	0.80	0.76	0.78
Cutoff Value^b^	0.68	0.57	0.58	0.57
Prevalence^c^	0.63		0.63	

Both models were developed using markers obtained from the 1^st^ biopsy only (median 9-days post-transplant) and markers from all biopsies obtained during the first 3 months to predict the risk of developing CAV at 5- or 10-years post-operatively.

**Abbreviations**: CAV: cardiac allograft vasculopathy; PPV: positive predictive value; NPV: negative predictive value.

a Pct correct: The percentage of cases correctly classified by the model considering both positive and negative classifications.

b Cutoff value: The predicted value from the logistic regression that serves as the threshold for predicting CAV. Patients with predicted values exceeding the cutoff are predicted to develop CAV.

c Prevalence: The proportion of patients that developed CAV during the indicated time interval.

**Table 9:** GFDCAV: Performance of the Established Model (which uses atherothrombotic markers, only), with the New Model (which adds inflammatory markers to the Established Model).

	Using 1^st^ biopsy, only		Using all 3-month biopsies	
***5-year risk***	*Established Model*	*New Model*	*Established Model*	*New Model*
Sensitivity	0.83	1.00	0.94	1.00
Specificity	0.85	0.80	0.82	0.84
PPV	0.39	0.35	0.39	0.41
NPV	0.98	1.00	0.99	1.00
Pct Correct^a^	0.85	0.82	0.84	0.85
Cutoff Value^b^	0.04	0.03	0.05	0.06
Prevalence^c^	0.10		0.10	
***10-year risk***				
Sensitivity	0.87	0.93	0.77	0.76
Specificity	0.74	0.75	0.89	0.90
PPV	0.43	0.46	0.60	0.65
NPV	0.96	0.98	0.95	0.95
Pct Correct^a^	0.77	0.78	0.87	0.88
Cutoff Value^b^	0.12	0.13	0.23	0.33
Prevalence^c^	0.18		0.18	

Both models were developed using markers obtained from the 1^st^ biopsy only (median 9-days post-op) and markers from all biopsies obtained during the first 3 months to predict the risk of graft failure at 5- or 10-years post-operatively.

**Abbreviations**: GFDCAV: graft failure due to cardiac allograft vasculopathy; PPV: positive predictive value; NPV: negative predictive value.

a Pct correct: The percentage of cases correctly classified by the model considering both positive and negative classifications.

b Cutoff value: The predicted value from the logistic regression that serves as the threshold for predicting GFDCAV. Patients with predicted values exceeding the cutoff are predicted to experience GFDCAV.

c Prevalence: The proportion of patients that had GFDCAV during the indicated time interval.

## Discussion

We have demonstrated that early inflammatory status is associated with long-term outcome following heart transplantation. Elevated serum CRP concentration, even when measured very early after transplantation, is a significant *independent* predictor of long-term CAV and GFDCAV. Inclusion of information about serum CRP, but not IL-6, improves the discriminative accuracy of the previously Established Models that use only AT risk predictors.

We have previously shown that when heart-transplant recipients exhibit early loss of microvascular antithrombin [8] and tPA [9], the presence of myocardial fibrin deposits [6], [7], and the expression of arterial endothelial ICAM-1 [3]–[5] in biopsied heart tissue, they are more likely to develop long-term CAV and GFDCAV in the ensuing 10 years. Recently, we developed multivariate prediction models that combine information from these AT markers (the Established Models in this paper). Here, for the first time, we have incorporated inflammatory markers into those Established Models and have shown that the New Models possess better discriminative accuracy and enhanced ability to classify patients correctly.

Although AUROC statistics improved in all cases when the New and Established Models were compared, some of the differences were small and not all were statistically significant. It is well known that when the AUROCs of two models (one with and one without the new marker) are compared, the difference is often small [17] and large odds ratios are required to cause a meaningful increase [18]. Additionally, AUROCs for our Established Models were already high, creating a ceiling effect that left very little margin for improvement.

The observation that CRP was a significant risk predictor in almost all cases, while IL-6 was not, may indicate a statistical correlation between these markers. Once one inflammatory marker explains the risk attributable to inflammation, there may be very little additional risk to be explained by a second inflammatory marker. Additionally, because IL-6 is a precursor of CRP, the presence of IL-6 may be *necessary* but not *sufficient* for elevated risk. In other words, risk might not be influenced by the presence of IL-6 alone, but only by the presence of IL-6 when it elicits CRP.

Our findings support the theory that CAV is a disease that is associated with endothelial activation and coagulation, as well as the increased presence of circulating inflammatory molecules such as CRP. From a risk-prediction perspective, these two processes appear to be statistically independent since adding inflammatory markers to our Established (atherothrombotic) Model improved prediction. But we have also shown that prediction is possible using inflammatory markers by themselves. A comparison of the AUROC values in **Tables 2** **and** **3** and **Fig. 2** reveals that models using inflammatory markers alone had nearly the same discriminative accuracy as the Established Models. This is a potentially important clinical finding that warrants further exploration, since it may be possible to develop good prediction models based solely on markers of systemic inflammation that can be measured frequently and inexpensively by a simple blood test. This is in contrast with atherothrombotic markers that can be determined only infrequently, are more expensive, and require an invasive biopsy.

From a larger scientific perspective, our findings suggest the potential utility of studying the transplanted heart as an accelerated model of native CAD. Although CAD takes a lifetime to develop in the general population, it develops analogously in the heart transplant patient over a span of months. Consequently, it may be possible to study the etiology of this disease prospectively, to identify biomarkers that predict its occurrence and course, and to test preventive interventions much more rapidly in cohorts of heart transplant patients than by observing the natural history of CAD in the general population.

Our study evaluated the statistical properties of IL-6 and CRP as biomarkers and early predictors of atherothrombosis. It was not designed to explain basic biological mechanisms. Our findings are consistent, however, with a growing body of biological evidence from recent experimental studies, however, revealing some of the causal relationships that may exist between proinflammatory mediators of atherothrombosis.

We have shown a strong association between a) CRP levels and endothelial activation, and b) CRP levels and subsequent development of CAV and graft failure [3]. CRP is known to be an important risk factor for native atherosclerosis and native CAD [19]. Elevated CRP plasma levels predict cardiovascular events among apparently healthy men [20]–[23] and women [24], patients with stable and unstable angina [25]–[29], and patients with a previous history of myocardial infarction [30]. Elevated CRP serum levels may promote atherosclerosis through its effect on adhesion molecule expression, since it has been shown that CRP induces ICAM-1 expression in coronary artery endothelial cells [31], [32]. Proinflammatory molecules, such as CRP can also down regulate tissue plasminogen activator (tPA) [33]. Possible links between CRP and adhesion molecule expression and between CRP and atherosclerosis have been reported, and an association has been demonstrated between elevated CRP levels and development of CAV [3] and graft failure in heart transplant recipients [3], [34].

IL-6 has been shown to directly promote coagulation, apparently without affecting fibrinolysis [35]. Cytokines such as interleukin-1, tumor necrosis factor, and IL-6 are also known to induce tissue factor expression in endothelial cells and circulating monocytes [36], [37]. Moreover, fibrin and fibrin degradation induce expression of proinflammatory cytokines such as IL-6 and interleukin-8 [36]. This cross-stimulation can lead to the persistence of a proinflammatory and prothrombotic microenvironment in the allograft. IL-6 also induces ICAM-1 expression on endothelial cells [38], [39] and may play a role in development and progression of atherosclerosis; adhesion molecules such as ICAM-1 and VCAM-1 play a significant role in cell recruitment within the intima during atheroma formation [31].

Another marker of inflammation, CRP, is mainly released by hepatocytes after IL-6 stimulation and is independently predictive of atherothrombosis. CRP is also associated with increased endothelial activation, soluble ICAM-1 levels [3], [40], and angiographic evidence of atherothrombosis development and progression [12], [40]. Patients who develop atherothrombosis have significantly higher levels of IL-6 and CRP compared to the patients who do not develop atherothrombosis [12], [40], [41]. Importantly, these same markers are predictive of CAD and cardiovascular events in the general population. The significance of CRP as a predictor of atherosclerosis-related events, however, remains controversial [42].

Several investigators have evaluated the association of plasma CRP levels with CAV and cardiac graft survival. Pethig et al [12] suggested that progressive CAV is accompanied by elevated CRP levels. Labarrere and colleagues [3] demonstrated that early increases in CRP are associated with an increase in cardiac ICAM-1 expression and soluble ICAM-1 levels, which is predictive of more aggressive CAV and graft failure. Hognestad et al [40] suggested a link between CRP and CAV and also correlated statin therapy with a decrease in CRP levels, providing further evidence for the role of inflammation in CAV [43].

Our study has strengths and weaknesses. Weaknesses include the utilization of angiography rather than intravascular ultrasound [44] and the lack of baseline angiograms at the time of transplantation. From a statistical point of view, our prediction models ultimately need to be tested in other populations by other investigators working in other settings in order to evaluate their generalizability. However, our models did undergo cross-validation on repeated bootstrap samples. Cross-validation produces estimates of a model's likely performance on future data and greatly reduces the likelihood of spurious variable selection that is often the most important source of bias arising from stepwise regression on a single sample [45]. Strengths include the relatively large number of transplant patients, the long multi-year follow-up, and the availability of a large immunohistochemical heart-biopsy database.

This study, along with the results of our previous work [31] on atherothrombosis following heart transplantation, suggests a fascinating and still incompletely understood relationship between inflammatory molecules and atherothrombosis in the pathogenesis of CAV and its complications. The involvement of CRP in arterial vasculopathy of transplanted hearts points to the possibility of using these molecules as therapeutic targets [46] to block or delay disease development or progression.

From a clinical perspective, our findings suggest new possibilities for exploring a multifactor battery of biomarkers such as fibrin deposits within the graft microvasculature, increased serum cardiac troponin I levels as evidence of myocardial damage within the allografts, markers associated with a lack of anticoagulant and fibrinolytic capacity, and activation markers derived from the up-regulation of endothelial ICAM-1 [4]–[9], [47], [48], which had been demonstrated to be early biomarkers of a negative outcome. Furthermore, although high circulating levels of the pro-inflammatory molecule CRP, a major component of the innate immune system in humans, is associated with the development and progression of native atherosclerosis and CAV, another component of the innate immune system, immunoglobulin (Ig) M and/or IgG natural antibodies (NAbs), may convey an atheroprotective function since high titers of IgM and/or IgG NAbs have been associated with reduced atherosclerosis and low levels are associated with reduced vein graft plaque in mice [49] and reduced atherosclerosis in mice and humans [50]–[55]. NAbs could also be added as future biomarkers to evaluate the risk of CAV in the heart transplant population.

There are three important implications of our findings that are of practical significance for clinical practice.

First, heart transplant patients should be assessed for both (1) early signs of atherothrombosis, as represented in our previously described models [10], [11] by the presence of microvascular fibrin and the depletion of tPA, and (2) early signs of inflammation, as represented in the new models described herein by the presence of CRP and its precursor, IL-6. The present study shows that both atherothrombosis and inflammation are important because they are independently predictive. Knowing a patient's inflammatory status adds prognostic information beyond that which would otherwise be available from knowing only the patient's atherothrombotic status.

Second, valuable clinical predictions about a patient's long-term chances of developing CAV and GFDCAV can be made very early, within days of the transplant procedure. Our findings show that almost as much prognostic information can be gained from a single biopsy obtained immediately after transplantation as can be gained from multiple biopsies obtained over the first 3 months.

Third, the clinical significance of these early markers relates primarily to their ability to make *negative* predictions. A patient who has a profile immediately after transplantation that is characterized by low inflammation and an absence of microvascular atherothrombosis is very unlikely to develop CAV or graft failure over at least the next 10 years. These patients may be able to safely forgo intensive follow-up and may be able to avoid frequent invasive testing that not only increases costs for the health care system but also poses risks for the patient. On the other hand, these markers are less accurate when it comes to positive predictions. Knowing that a patient has tested positive for early inflammation and/or signs of atherthrombosis does not mean that the patient is inevitably destined to develop CAV. Continued testing is indicated for those who test positive. Finally, the study's sample size and focus on early results offer insight into outcomes for transplant patients. Other studies are needed to validate these findings and investigate whether the early outcomes' predictive abilities hold for longer follow-up times.
